# Validation in the General Population of the iHealth Track Blood Pressure Monitor for Self-Measurement According to the European Society of Hypertension International Protocol Revision 2010: Descriptive Investigation

**DOI:** 10.2196/13137

**Published:** 2019-03-19

**Authors:** Victoria Mazoteras-Pardo, Ricardo Becerro-De-Bengoa-Vallejo, Marta Elena Losa-Iglesias, Daniel López-López, Patricia Palomo-López, David Rodríguez-Sanz, César Calvo-Lobo

**Affiliations:** 1 Facultad de Enfermería, Fisioterapia y Podología Universidad Complutense de Madrid Madrid Spain; 2 Faculty of Health Sciences Universidad Rey Juan Carlos Alcorcón Spain; 3 Research, Health and Podiatry Unit Department of Health Sciences, Faculty of Nursing and Podiatry Universidade da Coruña Ferrol Spain; 4 University Center of Plasencia Universidad de Extremadura Plasencia Spain; 5 Faculty of Sports Universidad Europea de Madrid Villaviciosa de Odón Spain; 6 Nursing and Physical Therapy Department Institute of Biomedicine, Faculty of Health Sciences Universidad de León Ponferrada Spain

**Keywords:** blood pressure determination, heart rate determination, validation studies, telemedicine

## Abstract

**Background:**

High blood pressure is one of the most common reasons why patients seek assistance in daily clinical practice. Screening for hypertension is fundamental and, because hypertension is identified only when blood pressure is measured, accurate measurements are key to the diagnosis and management of this disease. The European Society of Hypertension International Protocol revision 2010 (ESH-IP2) was developed to assess the validity of automatic blood pressure measuring devices that are increasingly being used to replace mercury sphygmomanometers.

**Objective:**

We sought to determine whether the iHealth Track blood pressure monitor meets ESH-IP2 requirements for self-measurement of blood pressure and heart rate at the brachial level and is appropriate for use in the general population.

**Methods:**

This study was a descriptive investigation. ESH-IP2 requires a total number of 33 participants. For each measure, the difference between observer and device blood pressure and heart rate values is calculated. In all, 99 pairs of blood pressure differences are classified into 3 categories (≤5, ≤10, and ≤15 mm Hg), and 99 pairs of heart rate differences are classified into 3 categories (≤3, ≤5, and ≤8 beats/min). We followed these protocol procedures in a convenience sample of 33 participants.

**Results:**

iHealth Track fulfilled ESH-IP2 requirements and passed the validation process successfully. We observed an absolute difference within 5 mm Hg in 75 of 99 comparisons for systolic blood pressure, 78 of 99 comparisons for diastolic blood pressure, and 89 of 99 comparisons for heart rate. The mean differences between the test and standard readings were 4.19 (SD 4.48) mm Hg for systolic blood pressure, 3.74 (SD 4.55) mm Hg for diastolic blood pressure, and 1.95 (SD 3.27) beats/min for heart rate. With regard to part 2 of ESH-IP2, we observed a minimum of 2 of 3 measurements within a 5-mm Hg difference in 29 of 33 participants for systolic blood pressure and 26 of 33 for diastolic blood pressure, and a minimum of 2 of 3 measurements within a 3-beat/min difference in 30 of 33 participants for heart rate.

**Conclusions:**

iHealth Track readings differed from the standard by less than 5, 10, and 15 mm Hg, fulfilling ESH-IP2 requirements. Consequently, this device is suitable for use in the general population.

## Introduction

### Background

High blood pressure (BP) is one of the most common reasons why patients seek assistance in daily clinical practice [1-7]. In addition, patients who have normal BP levels at age 50 years have a 90% lifetime risk of developing hypertension [8,9]. Hypertension is the second most frequent cause of cardiovascular diseases, which are the main cause of morbidity and mortality in the world [4,8,9]. Thus, BP maintains a strong, continuous, gradual, consistent, predictive, and independent relationship with the appearance of serious cardiovascular complications, such as peripheral arterial disease, stroke, heart attack, sudden death, or heart failure, or with renal pathologies.

Screening for hypertension is therefore fundamental, and many interventions are available, both pharmacologic and nonpharmacologic, to control such pathology and its consequences [3,5,6]. Because hypertension is identified only when BP is measured [10], accurate measurements are key to the diagnosis and management of this disease [11-13].

Mercury sphygmomanometers are being used less and less due to prohibitions against the use of mercury. Automatic BP devices are therefore replacing mercury sphygmomanometers [13-15].

To assess the validity of these automatic BP devices, 3 different protocols are used: that of the Association for the Advancement of Medical Instrumentation (AAMI) [16], that of the British Hypertension Society [17], and that of the Working Group on Blood Pressure Monitoring of the European Society of Hypertension (ESH) [18], which combined the previous protocols. The ESH protocol was revised in 2010 (ESH-IP2) [19] to be more demanding than the previous one. These 3 protocols are for adults in the general population.

### Objectives

The purpose of this study was to validate an automatic monitor that measures BP, iHealth Track, in the general population, following ESH-IP2 [19].

We hypothesized that the iHealth Track for home BP monitoring would show validated measures of BP and heart rate (HR), and would meet the requirements of ESH-IP2 in the general population.

## Methods

### Study Design

This study was a descriptive investigation to validate the iHealth Track device for the measurement of BP in the general population according to ESH-IP2 [19] and the Strengthening the Reporting of Observational Studies in Epidemiology criteria [20].

### Ethical Information

The Clinical Research Ethics Committee of Hospital Clínico San Carlos in Madrid, Spain, approved this study (number 18/504-O_P).

This study complies with the ethical principles of the Declaration of Helsinki [21], including amendments from 2000 to 2013.

All participants were informed about the study and gave written informed consent to participate.

### The Devices

#### Omron M3 IntelliSense

The reference device used in the study was the Omron M3 IntelliSense (Omron Healthcare, Inc, Kyoto, Japan), which was validated according to the ESH-IP2 [22]. It consists of an automatic oscillometric sphygmomanometer for the automatic measurement of BP and HR at the arm. The device has 2 available sleeve sizes: a standard size that fits arm circumferences of 22 to 32 cm and a larger size for arm circumferences of 32 to 42 cm.

#### iHealth Track

The device to be validated was the iHealth Track automatic device (KN-550BT; iHealthLabs Europe, Paris, France), which records brachial BP with the oscillometric method, with a range of pressure of 0 to 300 mm Hg (measuring accuracy ±3 mm Hg) and an HR range of 40 to 180 beats/min (measurement accuracy ±5%).

The systolic BP (SBP), diastolic BP (DBP), and HR are displayed on a liquid crystal display screen. The device has enough memory for 99 measurements. In addition, this unit can be used with Apple Bluetooth 4.0 devices and certain Android Bluetooth 4.0 mobile phones, by means of an app called Health MyVitals, which means that it allows for storage of BP and HR data in wireless devices connected to an iHealth Track and tracks data graphically and visually.

**Blood pressure (BP) and heart rate (HR) measurement protocol.**

- BPA: entry BP and HR, with the standard device (Omron M3 IntelliSense).
- BPB: device detection of BP and HR with the test instrument (iHealth Track). This measurement is used to determine the correct functioning of the test instrument with the participant and is discarded from further analysis.
- BP1: with the standard device.
- BP2: with the test instrument.
- BP3: with the standard device.
- BP4: with the test instrument.
- BP5: with the standard device.
- BP6: with the test instrument.
- BP7: with the standard device.

The unit weighs approximately 348 g (batteries and sleeve included). It required 4 AAA batteries with an approximate capacity of 250 measurements. The included standard cuff fits the circumferences of the arm in a range of 22 to 42 cm.

### Participants and Recruitment

We recruited a convenience sample of participants in Plasencia, Spain, from a family medical practice known to one of us.

According to the protocol review [19], we included 33 evaluable participants in the study who met the selection criteria, that is, all the inclusion and none of exclusion criteria. The inclusion criteria were men and women of at least 25 years of age, of whom at least 10 were men and 10 were women. The exclusion criteria were having a sustained arrhythmia, circulatory problems contraindicating the use of the cuff, or being pregnant.

### Study Protocol

The validation team consisted of a single researcher with experience in the measurement of BP.

The measurement room was at a comfortable temperature and without any factors that could influence the measurements, including noise and distractions [18,19].

Each participant reported his or her sex and date of birth. We registered weight, height, and body mass index (BMI, calculated using the Quetelet index, where BMI = weight in kilograms / height in meters squared) and measured the arm circumference to ensure that the cuff size was adequate. Subsequently, the participant relaxed for 10 minutes and 9 consecutive BP measurements were taken on the same arm, with the left arm at heart level, according to ESH-IP2 [18,19]. Measurements were taken alternating the Omron M3 IntelliSense (5 times) and the iHealth Track (4 times), as Textbox 1 outlines.

During measurement, participants remained calm, quiet, sitting, and not moving, with the back straight, keeping the feet on the floor in a parallel position, without crossing the legs. They rested the arm on a flat surface, with the palm of the hand upward and the elbow slightly flexed so that their arm was at the height of the heart. The interval between BP measurements was 30 to 60 seconds [19].

All measurements were made in the same room.

### Data Analysis

We performed statistical analyses using IBM SPSS Statistics, version 19 (IBM Corporation). The results are expressed as mean (SD). According to the normality tests of the Shapiro-Wilk test, we analyzed nonparametric data by the Wilcoxon-Mann-Whitney test and parametric data by means of the Student *t* test for independent samples. Statistical significance was set at *P*<.05.

We based the accuracy of a device, according to ESH-IP2, on comparisons between the test device (iHealth Track) and the reference device (Omron M3). For each participant, we first compared the device measurements BP2, BP4, and BP6 with the standard measurements BP1, BP3, and BP5, respectively, and then with the standard measurements BP3, BP5, and BP7, respectively. We classified the differences between these 2 devices separately for both SBP and DBP according to whether their values were within 5, 10, or 15 mm Hg [19] and, for HR, according to whether their values were within 3, 5, or 8 beats/min.

We analyzed and expressed results according to ESH-IP2 requirements to determine whether the device passed or failed the validation protocol. Part 1 and part 2 of the validation process concern the number of differences in the requested ranges for individual measurements (99 measurements) and for individual participants (33 participants), respectively [19].

We used Bland-Altman plots to represent the relationship of the SBP difference (device reference) and mean SBP (device and reference); DBP difference (device reference) and mean DBP (device and reference); and HR difference (device reference) and mean HR (device and reference).

## Results

### Participants

We screened a total of 33 participants. There were 13 men and 20 women. Table 1 shows their age, weight, height, BMI, and arm circumference, as well as the comparison of these according to sex.

**Table 1:** Sociodemographics characteristics of the participants.

Characteristics	Total group (N=33)		Men (n=13)		Women (n=20)		*P* value
	Mean (SD)	Range	Mean (SD)	Range	Mean (SD)	Range	
Age (years)	47.94 (17.21)	25-87	45.85 (16.66)	30.0-84.0	49.30 (17.85)	25.0-87.0	.58^a^
Weight (kg)	72.45 (10.47)	54-92	75.54 (9.10)	61.0-90.0	70.45 (11.02)	54.0-92.0	.18^b^
Height (cm)	167.06 (5.51)	158.0-178.0	171.0 (3.48)	165.0-178.0	165.0 (5.16)	158.0-175.0	.001^b^
Body mass index (kg/m^2^)	25.88 (2.85)	20.32-31.14	25.80 (2.43)	22.41-29.39	25.93 (3.15)	20.32-31.14	.9^b^
Arm circumference (mm)	285.76 (21.80)	230.0-320.0	293.08 (13.76)	260.0-310.0	281.0 (24.90)	230.0-320.0	.12^b^

^a^Nonparametric Wilcoxon-Mann-Whitney test.

^b^Parametric independent Student *t* test. *P*<.05 was considered statistically significant, with a confidence interval of 95%.

### BP Measurements

**Figure 1:**
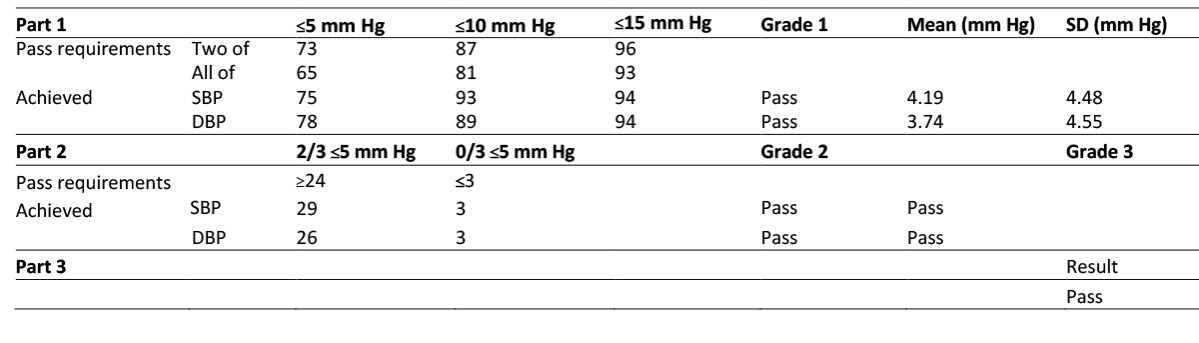
Validation results for the iHealth Track blood pressure device according to European Society of Hypertension International Protocol revision 2010 (ESH-IP2). Accuracy is determined by the number differences in these ranges both for individual measurements (Part 1) and for individual subjects (Part 2). To pass, a device must achieve all the minimum pass requirements shown. Pass requirements are as required by the EHS-IP2; achieved are as recorded by the device. DBP: diastolic blood pressure; SBP: systolic blood pressure.

Figure 1 shows the validation results for the iHealth Track BP device according to ESH-IP2. The table shows the numbers of measurements differing from the standard device, Omron M3, by 5, 10, and 15 mm Hg or less, for SBP and DBP, according to ESH-IP2 [19]. The mean differences between the standard device and the tested device were 4.19 (SD 4.48) mm Hg for SBP and 3.74 (SD 4.55) mm Hg for DBP.

These analyses showed an absolute difference within 5 mm Hg in 75 of 99 pairs of differences for SBP and in 78 of 99 pairs for DBP (vs at least 73 for SBP and 65 for DBP following ESH-IP2 requirements). We observed an absolute difference within 10 mm Hg in 93 of 99 comparisons for SBP and in 89 of 99 comparisons for DBP (vs at least 87 for SBP and 81 for DBP following ESH-IP2 requirements). Additionally, 94 of 99 comparisons exhibited an absolute difference within 15 mm Hg for SBP an DBP (vs at least 96 for SBP and 93 for DBP following ESH-IP2 requirements). Therefore, we successfully completed part 1 of the device validation for BP.

For part 2 of ESH-IP2, 29 of 33 individuals had a minimum of 2 of 3 comparisons within a 5-mm Hg difference for SBP, and 26 of 33 participants met this requirement for DBP (vs at least 24 of 33 participants for SBP and DBP following ESH-IP2 requirements). On the other hand, 3 comparisons exceeded the 5 mm Hg requirement for SBP and DBP in 3 of 33 participants (vs a maximum of 3 participants for SBP and DBP following ESH-IP2 requirements). Because these 2 conditions were validated, we successfully completed part 2 of the device validation for BP.

Thus, part 3 of the iHealth Track device validation also was passed, because parts 1 and 2 were both validated for SBP and DBP.

**Figure 2:**
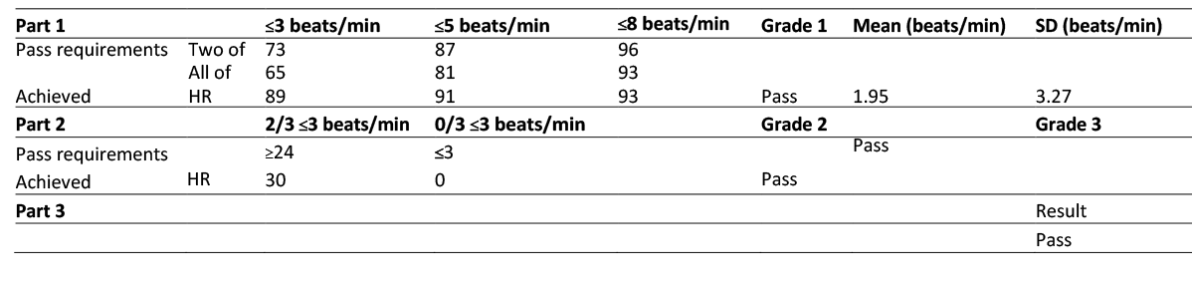
Validation results for the iHealth Track heart rate (HR) device according to European Society of Hypertension International Protocol revision 2010 (ESH-IP2). Accuracy is determined by the number differences in these ranges both for individual measurements (Part 1) and for individual subjects (Part 2). To pass, a device must achieve all the minimum pass requirements shown. Pass requirements are as required by the EHS-IP2; achieved are as recorded by the device.

Figure 2 shows the validation results for the iHealth Track HR device according to ESH-IP2. The numbers of measurements differing from the standard Omron M3 device by 3, 5, and 8 beats/min or less are reported for HR. The mean difference between the standard and the tested device was 1.95 (SD 3.27) beats/min.

These analyses showed that 89 of 99 comparisons had an absolute difference within 3 beats/min, 91 of 99 comparisons had an absolute difference within 5 beats/min, and 93 of 99 differences had an absolute difference within 8 beats/min. Therefore, we successfully completed part 1 of the device validation for HR.

For part 2 of ESH-IP2, 30 of 33 individuals had a minimum of 2 of 3 comparisons within a 3-beats/min difference for HR. On the other hand, none of the 33 participants had 3 differences exceeding 3 beats/min. Because these 2 conditions were validated, we successfully completed part 2 of the device validation for HR.

Thus, part 3 of the iHealth Track device validation was passed, because parts 1 and 2 were both validated for HR.

With these results, the iHealth Track device meets ESH-IP2 validation criteria for both BP (SBP and DBP) and HR for use in the general population.

**Figure 3:**
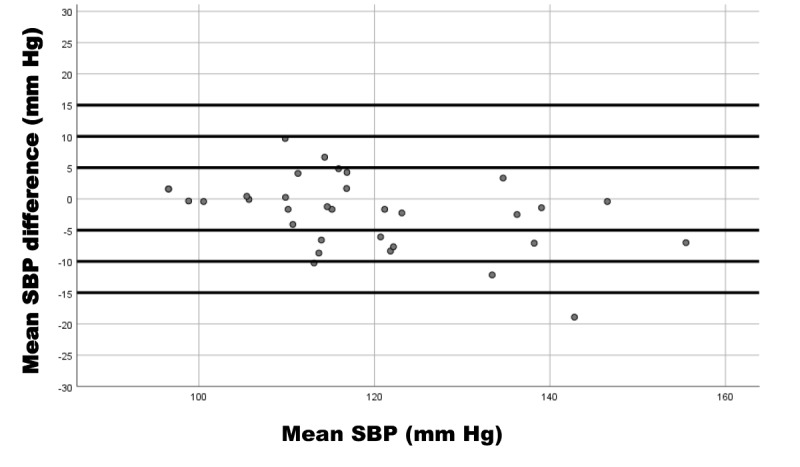
Bland-Altman plot of systolic blood pressure (SBP) measurement differences between the iHealth Track (test) and the Omron M3 (reference) devices in 33 participants. Mean SBP difference is the systolic difference between the devices; mean SBP is the mean systolic average values of the devices.

**Figure 4:**
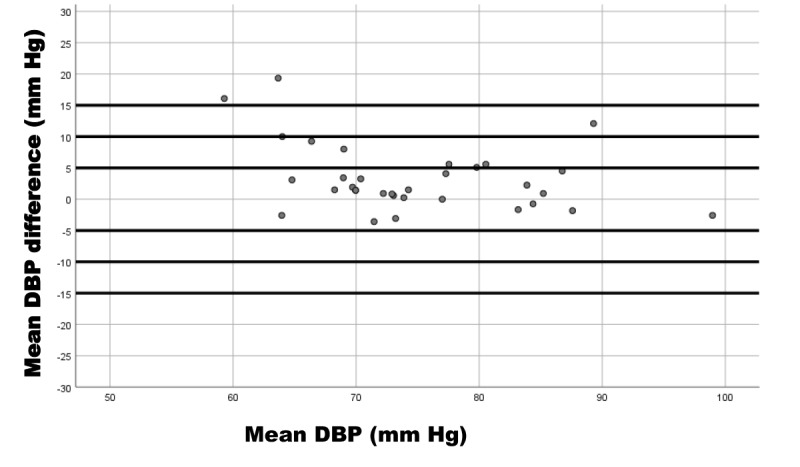
Bland-Altman plot of diastolic blood pressure (DBP) measurement differences between the iHealth Track (test) and the Omron M3 (reference) devices in 33 participants. Mean DBP difference is the diastolic difference between the devices; mean DBP is the mean diastolic average values of the devices.

**Figure 5:**
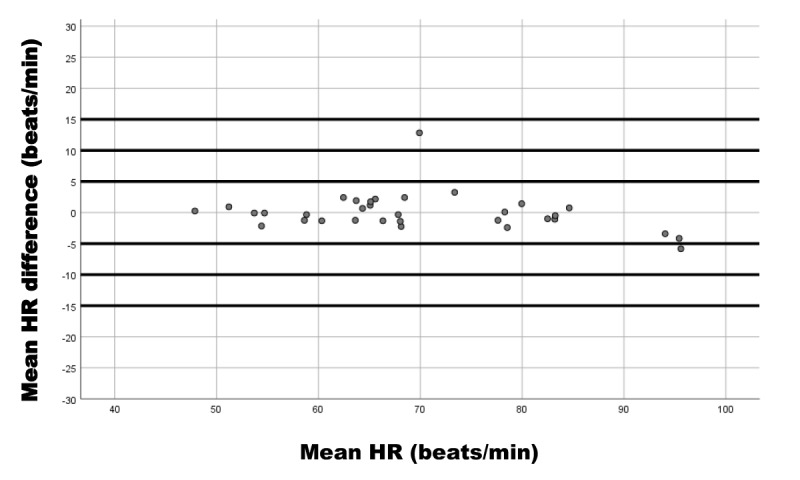
Bland-Altman plot of heart rate (HR) measurement differences between the iHealth Track (test) and the Omron M3 (reference) devices in 33 participants. Mean HR difference is the heart rate difference between the devices; mean HR is the mean heart rate average values of the devices.

These results coincide with the Bland-Altman plots showing the differences in measurements between the iHealth Track device and the Omron M3 for SBP (Figure 3), DBP (Figure 4), and HR (Figure 5).

## Discussion

### Principal Results

The results obtained from this research are important because they are the first to show that the iHealth Track device successfully passed both parts of the validation process according to the ESH-IP2 review in the general population [19]. However, these results cannot be extrapolated to other specific populations, such as older, diabetic, or pregnant individuals, because we have not addressed these conditions.

Regarding the validation protocols, the presence of several protocols for this function [16-19] is problematic for several reasons. Manufacturers cannot perform the 3 protocols and experts focus on their own protocols (eg, the AAMI is followed more in the United States and the ESH is followed in Europe), and it is impossible to compare several validation studies that are governed by different principles [23].

### Limitations

In this study, we used the ESH protocol, first published in 2002 [18] and revised in 2010 [19], which has many advantages over previous ones [16,17], but also has some limitations.

First, ESH-IP2 does not specify the number of validation studies needed to validate the instrument, although a few findings suggest that a device should be validated in no fewer than 2 different centers separately [22-24]. In this regard, the protocol of the AAMI recommends conducting more than 1 study but does not specify the number of studies or devices [16]. Therefore, it is important to check the validity of BP measurement devices before widespread application in clinics and homes.

Second, the specific conditions required for the participants recruited in the study exclude children and young people, thus omitting data on the hypertensive population between 18 and 25 years of age.

Third, although we calculated the sample size, consecutive sampling bias should be considered, and a simple randomization sampling process could be more adequate for future studies.

Fourth, ESH-IP2 mentions no explicit criteria for a validation process in specific populations, and we highly recommended that this be taken into consideration in its next revision.

Fifth, although sphygmomanometers measure SBP, DBP, and HR, no version of the international protocol of the ESH considers validating HR. Hence, we have added such a validation based on the protocol criteria in BP and establishing, in this case, the required differences based on the scale of values found after HR measurements, being even more demanding than the ESH.

### Conclusion

The results of this study are relevant because they show that an automatic wireless device that measures BP and HR and that can also be linked to new technologies meets the requirements of ESH-IP2.

We highly recommended that the accuracy of iHealth Track be assessed in other specific populations, such as pregnant women, older people, patients with arrhythmia, and so on, using other types of sampling. Also, it would be convenient to extend the validation equipment in order to reduce intraobserver error.

## Abbreviations

AAMI: Association for the Advancement of Medical Instrumentation
BP: blood pressure
DBP: diastolic blood pressure
ESH: European Society of Hypertension
ESH-IP2: European Society of Hypertension International Protocol revision 2010
HR: heart rate
SBP: systolic blood pressure
